# Would Altruistic Consumers Place A Higher Value on Sustainable Foods?

**DOI:** 10.3390/foods12193701

**Published:** 2023-10-09

**Authors:** Haoyang Li, Wen Lin

**Affiliations:** 1 College of Economics and Management, Nanjing Agricultural University, Nanjing 210095, China; lihy@njau.edu.cn; 2 China Academy for Rural Development, School of Public Affairs, Zhejiang University, Hangzhou 310058, China

**Keywords:** sustainable labels, coffee, altruism, valuation

## Abstract

To advance sustainable food systems, it is necessary to explore consumer preferences and valuations of sustainable food labels. This study utilizes a discrete choice experiment to examine consumers’ willingness to pay for various sustainable labels using a 12 oz ground coffee package and investigates the impact of altruism orientation on consumer valuation of sustainable coffee. The results from US consumers surveyed in spring 2020 indicate that the USDA organic claim commands the highest price premium, followed by the Carbon Trust and Fairtrade labels. Furthermore, individuals’ altruism orientation positively and significantly influences their preference and willingness to pay for sustainable labels, with selfless individuals valuing them more. These findings offer insights into effectively promoting sustainable food consumption through targeting consumer subgroups and prioritizing fair trade and organic foods over newer green labels.

## 1. Introduction

Sustainable development poses a significant challenge to human society, and as a response, more than 190 countries signed the Paris Agreement in December 2015 to address climate change mitigation. Recently, achieving carbon neutrality has emerged as one of the world’s most pressing goals, with around 110 countries committing to reducing carbon dioxide emissions. Unfortunately, while considerable efforts have been dedicated to non-food sectors and recently some food sectors [1], the role of consumers in promoting sustainable food systems has received relatively less research attention in the literature. This is concerning considering that the food system is responsible for one third of global anthropogenic greenhouse gas emissions, as highlighted by Crippa et al. [2]. Therefore, promoting sustainable food systems is not only crucial but also essential for attaining sustainable development goals.

Sustainable labels have emerged as a promising tool for decarbonizing and enhancing the sustainability of food systems. Through providing information, these labels can empower individuals to make environmentally friendly food choices and incentivize the supply side to offer greener options in the market [3]. While previous studies have predominantly focused on the adoption behavior of green technologies by the supply side [4,5,6], such as food producers, plants, and retailers, there is a growing body of literature that explores the feasibility of promoting sustainable labels among consumers. These studies assess consumer preferences and valuations of different sustainable food products, examining individuals’ willingness to pay for various green labels, including organic and Fairtrade labels [7,8,9,10,11,12,13], animal welfare claims [14,15,16], carbon footprint labels [17,18,19,20], and the forage–livestock balance label [21], among others. While several studies have examined individual sustainable labels or compared a limited number of labels [22,23], there is a dearth of research that combines multiple sustainable labels within a single context and evaluates consumers’ willingness to pay for each label. Understanding the relative market value placed on different sustainable labels and the extent to which consumers value each of them can provide valuable insights for the supply side when considering and planning the adoption of sustainable labels. Conducting research to investigate the market’s preferences and valuations of various sustainable labels would contribute to filling this research gap. Such investigations would shed light on which sustainable label(s) are most or least valued by the market and provide valuable information for supply side actors in their decision-making processes regarding the adoption of sustainable labels.

In addition to understanding the factors that influence individual preferences and willingness to pay for sustainable labels, it is crucial to explore various elements in enhancing sustainable food consumption. Existing literature has explored factors such as demographics [24,25], beliefs [26], food consumption habits [27,28], and time preferences [29,30]. However, one aspect that has received comparatively less attention in the literature is altruism. Altruism, which refers to selfless concern for the well-being of others [31], can also have a significant influence on individuals’ attitudes toward sustainable labels and their willingness to pay for sustainable food products. Altruistic individuals often prioritize the welfare of others and the long-term health of the planet, and these individuals may be willing to pay a premium for sustainable food products that have the appropriate labels. They see their purchasing decisions as a means to support sustainable practices, promote fair trade, reduce carbon emissions, and encourage responsible resource management. On the other hand, some studies have demonstrated a positive association between altruism and a series of sustainable behaviors [32,33], including the purchases of local foods [34] and organic foods [35]. This implies the effect of altruism is likely to be evident in other contexts of food purchases.

Understanding the impact of altruism on consumers’ attitudes towards sustainable labels and food choices is therefore crucial for developing effective strategies to promote sustainable food consumption. Through considering individuals’ altruistic orientation, policymakers and marketers can tailor interventions and communication strategies to resonate with different altruistic attitudes, thereby encouraging the adoption of sustainable food options. To fill the gap, this study assesses consumers’ willingness to pay for different sustainable labels and the role that altruism plays in explaining consumer valuation of sustainable labels, with a discrete choice experiment of a package of 12 oz ground coffee conducted among U.S. consumers. Our study contributes to the literature in several ways. First, we investigate and compare the willingness to pay values for three sustainable labels, namely, organic, Fairtrade, and Carbon Trust labels, on food products under one study. Second, we explain consumers’ preferences and valuations of the three green labels with their altruism orientation.

## 2. Materials and Methods

### 2.1. Choice Experimental Design

To assess the value that US consumers place on sustainable food products, we devised and executed a hypothetical online choice experiment. The experiment involved presenting individuals with various decision scenarios or tasks, in which they were asked to select their preferred product option. Each scenario included multiple alternatives of the product, along with a no-purchase option, to simulate real-world market situations. In this study, we focused on a specific product: 12 oz roasted ground coffee, which is a commonly available item in the US market. Through selecting a well-known product, we aimed to ensure familiarity and relevance to participants, enhancing the realism of their decision-making process within the experiment.

Our choice experimental design incorporates four key attributes: USDA organic, Fairtrade, and Carbon Trust labels, as well as price. These attributes were carefully chosen to capture different dimensions of sustainability in the context of coffee products. The USDA organic, Fairtrade, and Carbon Trust labels serve as indicators of sustainable coffee products. Each of these attributes is represented as a binary variable, taking a value of one if the coffee product carries the respective label and zero otherwise. The organic and Fairtrade labels are well-established sustainability certifications, while the Carbon Trust label represents a newer and emerging attribute in the marketplace. Coffee production is known to contribute significantly to carbon emissions within the food system [36] and the inclusion of the Carbon Trust label signifies a commitment by the company to reducing the carbon footprint associated with their coffee products. In addition to the sustainability labels, we also consider price as an attribute in our design. The selected price levels encompass both high-end and low-end prices commonly observed in the real market, ranging from USD 4.95 to USD 12.45. Through including a range of price points, we aim to capture the influence of price sensitivity on consumers’ decision-making process. For a comprehensive overview of the chosen product attributes and attribute levels, please refer to Table 1.

**Figure 1:**
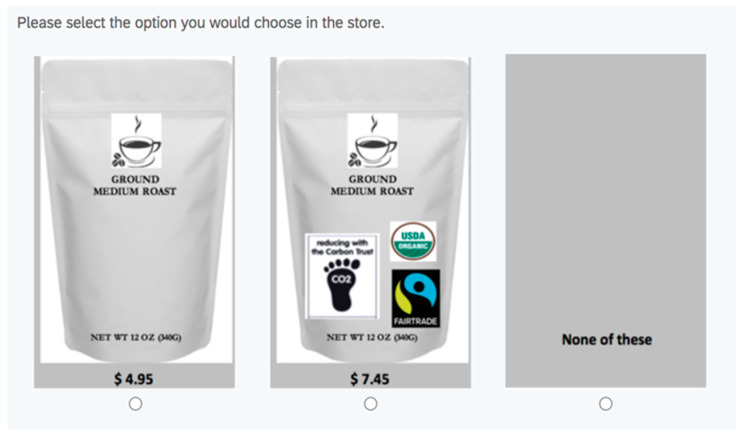
A sample choice set.

To ensure the efficiency and effectiveness of our choice experiment, we employed a D-efficient design methodology [37]. This approach allowed us to generate a total of 32 choice sets, organized into four blocks, with each block containing eight choice sets. The D-efficiency achieved in our design is an impressive 97.21%, indicating a high level of precision in capturing the relevant information from participants. Within each choice set, two product alternatives were presented, each with experimentally designed attribute levels. Additionally, a no-purchase or opt-out option was included to replicate real-market scenarios where consumers may choose not to make a purchase. To minimize any potential bias or order effects, participants were randomly assigned to one of the four blocks and completed eight choice tasks in a randomized order. This approach ensured a balanced distribution of choice sets across participants and reduced any potential systematic biases. For a visual representation, please refer to Figure 1, which showcases an actual choice set that was used in our experiment.

### 2.2. Altruism Measurement

To assess consumers’ altruism orientation, we employed the scale questions developed by Lusk et al. [38]. The psychometric scale consists of five questions listed in Table 2, and in each question, respondents were asked to indicate the extent to which they agree with the statement on a scale from 1 (strongly disagree) to 7 (strongly agree). Each statement is easy for respondents to understand and would not cause too much cognitive burden while eliciting their altruism orientation. As a standardized and efficient method for gaining insights into individual attitudes, beliefs, and preferences, multiple-item psychometric scales have been designed and used to quantify risk preferences, time preferences, and environmental beliefs, among others [26,29,39].

### 2.3. Analytical Framework

We employed a mixed logit model to analyze the data obtained from our choice experiment. The mixed logit model offers several advantages in estimating consumer preferences as it accommodates variations in consumer tastes related to unobserved variables. This flexibility enables the model to capture a more realistic substitution pattern among the available alternatives. Adhering to the principles of random utility theory, the utility of individual *n* selecting alternative *j* in choice situation *t* can be represented as follows:

(1) $$\begin{array}{c} U_{njt}=V_{njt}+\varepsilon_{njt}\\ =\alpha {Price}_{njt}+\beta_{n1}{Organics}_{njt}+\beta_{n2}{Fairtrade}_{njt}+\beta_{n3}{Carbtrust}_{njt}+{ASC}_{Opt-out}+\varepsilon_{njt} \end{array}$$

In the above equation, ${Price}_{njt}$ is a continuous variable that includes the four price levels used in the experimental design. ${Organics}_{njt}$, ${Fairtrade}_{njt}$, and ${Carbtrust}_{njt}$ are dummy variables indicating the presence (1) or absence (0) of USDA organic, Fairtrade, and Carbon Trust certifications, respectively, for the specific product. The coefficient α represents individual price sensitivity, while the β coefficients denote the taste coefficients associated with the non-price attributes. The term ${ASC}_{Opt-out}$ represents the alternative-specific constant for the opt-out option, which remains constant across the population. $\varepsilon_{njt}$ represents the random error term, assumed to follow a Type I Extreme Value distribution. To account for heterogeneity in individual preferences, the coefficients of the non-price product attributes are assumed to be random, following normal distributions. This allows for variations in how individuals value these attributes. The price coefficient (α), on the other hand, is specified as a fixed value.

Through considering these components, we can calculate the unconditional choice probability for individual *n* selecting alternative *j* in choice situation *t*:

(2) $$P_{njt}=\int_{\beta }^{}\frac{e^{V_{njt}}}{\sum_{k}e^{V_{nkt}}}f(\beta |\alpha ,{ASC}_{Opt-out},\bar{\beta },\Omega )d\beta$$

where $f(\beta |\alpha ,{ASC}_{Opt-out},\bar{\beta },\Omega )$ is the density function of the vector of random coefficients. The density function of the vector of random coefficients is determined by the parameters β and Ω, which represent the means and the variance-covariance matrix, respectively. These parameters govern the distribution of the random coefficients in the model. To estimate the model, we utilized simulated maximum likelihood estimation with 1000 Halton draws in STATA. This approach allows us to capture the variability in individual preferences and obtain reliable estimates of the model parameters. To compute the mean willingness to pay for the sustainable coffee label k for consumer n, we can use the estimated model. Consumer *n*’s mean willingness to pay for the sustainable coffee label *k* can be computed as:

(3) $${WTP}_{nk}=\frac{-\bar{\beta_{nk}}}{\alpha },\;k=1,\;2,\;3.$$

In our study, we examine and compare the willingness to pay values between groups that exhibit different time preferences. To achieve this, we match the altruism scores with individual-specific or conditional willingness to pay (WTP). Through assessing the relationship between altruism and WTPs, we aim to understand if there are significant differences in WTP based on individuals’ altruism orientation. To statistically test for WTP equality between these different groups, we employ the Poe test [40]. This statistical analysis helps us assess whether the observed differences in WTP between groups are statistically significant.

### 2.4. Data Collection and Quality Control

The survey was conducted in the spring of 2020 utilizing an online opt-in consumer panel sourced from Survey Sampling International. To be eligible for participation, individuals had to be at least 18 years old and have made coffee product purchases within the past two months. To ensure data integrity, respondents who could not commit to providing truthful answers, failed attention check questions or completed the survey outside the 95% confidence interval of the survey time were excluded from the analysis. Ultimately, a sample of 304 US respondents was collected, with the majority completing the survey within a 20 min timeframe.

The survey consisted of three parts: screening questions and the altruism scale questions, eight coffee choice tasks in the second part, and demographic inquiries in the final section. To enhance the reliability of survey responses, a “cheap talk” script was employed in an attempt to minimize potential hypothetical bias. Additionally, at the beginning of the survey, respondents were requested to commit to carefully reading the questions and providing honest answers. Those who could not make such a commitment were not included in the survey. Furthermore, two attention checks were incorporated into the questionnaire to identify and exclude participants who may have been inattentive. Through implementing these measures, we aimed to ensure the validity and accuracy of the collected data, thereby strengthening the robustness of our findings.

## 3. Results and Discussions

### 3.1. Sample Description

Table 3 displays the socio-demographic characteristics and choice frequencies of our sample. On average, respondents are around 51 years old, hold a college degree, have a household size ranging from 3 to 4 individuals, hold moderate political views, and have a pre-tax annual household income between 40,000 US to 80,000 US dollars. The majority of respondents identify as white and religious, and they mostly have part-time employment. This prevalence of part-time work may be attributed to the survey being conducted during the COVID-19 pandemic. Slightly over half of the sample consists of male participants. Despite the size of our sample, it can be considered broadly representative of the US population. Additionally, we provide an overview of the choices made by our respondents during the choice experimental tasks. Across the eight choice tasks, the average respondent abstains from purchasing any product alternative once, and they favor the more expensive product alternative in almost three out of the eight choice tasks. This suggests that the responses given by participants are reasonably consistent.

### 3.2. Preference and Willingness to Pay for Sustainable Coffee Labels and the Role of Altruism

We begin via examining the results in preference space and subsequently delve into the findings related to willingness to pay (WTP). The estimation results of the mixed logit models in preference space using STATA and 1000 Halton draws are presented in Table 4. The benchmark specification, Model (1), focuses on the main effects of product attributes. It includes the fixed coefficient for price taste and the parameter for non-price attributes, which follows a normal distribution. Model (2) builds upon Model (1) through incorporating interaction terms between altruism scores and product attributes. This addition allows us to investigate how altruism orientation influences individual preferences for sustainable food products.

Model (1) yields several noteworthy findings. Firstly, on average, individuals exhibit a preference for the three sustainable labels, with the USDA organic label being the most preferred, followed by the Carbon Trust label and the Fair Trade claim. Secondly, higher coffee prices result in a decrease in utility, aligning with economic principles. Thirdly, the parameter associated with the opt-out option is significantly negative (−5.3), indicating that individuals tend to choose coffee product alternatives over not purchasing any coffee products. Lastly, there is significant heterogeneity in individual preferences for the three sustainable labels, suggesting that not all participants favor sustainable coffee products, and some individuals even hold a negative valuation of these products. Approximately 34%, 39%, and 35% of respondents express a dislike for the USDA organic, Fair Trade, and Carbon Trust claims, respectively. Possible reasons for this disparity could be the widespread familiarity of the USDA organic and Fair Trade labels in the US market, while the Carbon Trust claim may be relatively novel for US consumers.

In order to better understand the observed heterogeneity in consumer preferences for sustainable labels, we investigate whether altruism preferences play a role in explaining this variation. This analysis is presented in Model (2) of Table 4. We observe a significant improvement in model fit through incorporating the interaction terms between the altruism preference scale and product attributes. This is evident from the increase in log-likelihood (from −1717 to −1684) and the decrease in AIC per person (from 11.35 to 11.19). Furthermore, the inclusion of the interaction terms in Model (2) leads to a reduction in the estimated standard deviations of product tastes, which reflects a decrease in unobserved taste heterogeneity. These statistical findings provide evidence that consumers’ preferences for sustainable food products are influenced by their altruism orientation, emphasizing the importance of considering altruism attitudes when studying consumer food choices. The coefficients of the interaction terms reveal several findings. First, altruism orientation significantly affects consumer choices of organic, Fairtrade, and Carbon Trust-labeled coffee products. Specifically, a person would have stronger preferences for these environmentally friendly coffee products if her altruistic inclination is stronger. This is expected because individuals with stronger altruistic tendencies prioritize the external impacts of their actions. Consequently, they are more inclined to choose products that contribute to broader benefits and positive outcomes beyond their personal interests. Second, among the three sustainable labels, consumer choices of Fairtrade labeled coffee are mostly explained by altruism, followed by the choices of USDA organic and Carbon Trust-claimed coffee products. An individual with an altruism score that is one point higher than the average is expected to derive an additional utility of 0.061 and 0.058 from selecting Fairtrade coffee and organic coffee, respectively. Altruistic individuals, on the other hand, tend to exhibit comparatively weaker preferences for Carbon Trust claims, and those whose altruism scale scored one point higher than the sample mean would have 0.029 higher utility than the average, with a 10% statistical significance level.

We now turn our attention to the willingness to pay (WTP) values derived from the preference-space model estimates in Model (1) of Table 4. Table 5 presents the average WTPs for sustainable labels in the full sample, as well as in four subsamples categorized according to altruism attitudes. In line with the findings presented in Table 4, the highest WTP is observed for organic coffee (USD 1.2), followed by Carbon Trust-labeled coffee (USD 0.75) and Fairtrade-labeled coffee (USD 0.73). On average, consumers in the United States are willing to pay an additional USD 1.2 for a 12 oz serving of organic coffee compared to unlabeled coffee products. When examining the WTPs for sustainable coffee products across different subsamples, we observe that individuals with lower altruism scores tend to assign lower price premiums for sustainable coffee. To illustrate this, compared to conventional coffee, the valuation of organic coffee is USD 1.48 and USD 1.0 higher per 12 oz in the above- and below-median altruism score subsamples, respectively; the valuation of Fairtrade-labeled coffee is USD 0.63 and USD 0.85 higher in the above- and below-median altruism score subsamples, respectively. Additionally, for the two WTP values, the difference between above and below-median score subsamples is significant at least at a 5% level. These findings reinforce the notion that individuals with higher levels of altruism are inclined to select sustainable products that benefit both others and the environment.

### 3.3. Discussions

Our study reveals that among the organic, Fairtrade, and Carbon Trust labels, US consumers display a preference for the organic label, followed by Fairtrade and Carbon Trust labels. Corresponding to this preference ranking, consumers are willing to pay an additional amount of USD 1.20, USD 0.75, and USD 0.73 for organic-, Fairtrade-, and Carbon Trust-labeled coffee, respectively, in comparison to conventional coffee. These values align with the findings of Van Loo et al. [13], who reported a willingness to pay USD 1.16 for organic coffee and USD 0.68 for Fairtrade coffee among US consumers. Regarding Carbon Trust labels, prior research suggests that their impact on Americans’ utility in food product selection is relatively weaker compared to the organic logo, antibiotic-free claims, and even conventional production methods [29,41]. Notably, the higher price premium associated with the organic label may be attributed to consumers’ perception of the health and environmental benefits associated with organic foods [38,42,43]. For example, Durham and Andrade [44] proposed that environmental concern holds more influence than health considerations in consumers’ decisions to purchase organic foods, specifically in the context of US consumer choices of fruits and vegetables.

Additionally, our study uncovers variations in consumer preferences and valuations of the three sustainable labels based on their altruistic orientation. The inclusion of interaction terms between the altruism score and the three labels proves statistically significant, indicating that incorporating altruism orientation in the analysis of food choices provides a better explanation for the heterogeneity surrounding random taste parameters and individuals’ decision-making. Expectedly, people with a stronger altruism orientation are more careful about sustainable labels and tend to pay more for them, particularly in terms of Fairtrade labels. Altruistic individuals prefer the Fairtrade label most for several reasons. Fair trade practices are often associated with ethical considerations and social responsibility [45]. Altruistic individuals tend to prioritize fairness, justice, and the well-being of others. Through choosing Fairtrade products, they can support producers in developing countries and contribute to reducing poverty and inequality in global supply chains. Secondly, Fairtrade certification ensures that producers receive fair prices for their goods, which can help improve their living standards and empower them economically. Altruistic individuals may be motivated to support fair trade as a means of promoting economic justice and sustainability in trade relationships. Thirdly, fair trade practices often involve environmentally sustainable production methods and community development initiatives. Altruistic individuals, who are concerned about the broader impacts of their consumption choices, may view fair trade as a way to contribute to environmental conservation and community empowerment. Our findings are in line with the studies suggesting that altruistic people tend to have more sustainable behaviors in the context of non-food consumption [32,46,47,48,49] as well as food purchases [34,50,51].

The findings of our study carry implications for advancing sustainability efforts from both the demand side and the perspective of food systems. While previous research has predominantly focused on the supply side, examining the adoption of green technologies by farmers or agricultural firms, it is essential to recognize that the development of sustainable food systems also heavily relies on consumer attitudes and preferences towards environmentally friendly foods. To promote increased sustainable food consumption, policymakers and food suppliers may need to adopt differentiated strategies that cater to the preferences of individuals with different altruistic preferences. For individuals with a strong altruistic orientation, it would be beneficial to highlight the public benefits of green consumption. Conversely, for selfish individuals, emphasizing the private benefits of sustainable food choices would further enhance their inclination toward sustainability. To effectively communicate such messages, the implementation of information provision and targeted marketing campaigns would be instrumental. These strategies can effectively educate consumers about the environmental and societal benefits associated with sustainable food choices, thereby encouraging and facilitating their adoption.

Our study identifies several potential areas for future research. Firstly, while our respondents were geographically diverse, the sample size of our study was relatively small. Future studies could address this limitation through examining our research questions using larger sample sizes and expanding to other contexts. This could include investigating developing countries that face significant environmental challenges and exploring the application of sustainable labels to other food products, such as red meat or even daily dietary choices. Secondly, we employed a hypothetical choice experiment due to the unavailability of Carbon Trust labels in the US market at the time of our study. Although we implemented various strategies to ensure response quality, such as an oath at the survey’s outset, a cheap talk script before choice tasks, and attention trap questions, it is important to acknowledge the potential impact of hypothetical bias on our results. Future research could address this issue through investigating individual preferences and willingness to pay for sustainable labels using real experiments or market transaction data once all the sustainable labeled products are available in the market. Thirdly, our study highlights the significance of behavioral factors, beyond monetary mechanisms, in influencing consumers’ food choices. It is worth exploring other behavioral factors, such as personality traits and cognitive biases, in future studies to gain a more comprehensive understanding of consumer preferences.

## 4. Conclusions

This research employed a discrete choice experiment to investigate the willingness to pay of US consumers for various sustainable labels on 12 oz ground coffee, while also exploring the role of altruism in shaping consumer preferences for sustainable labels. The study reveals that among the three sustainable labels examined, the USDA organic claim commands the highest price premium compared to unlabeled and conventional coffee, followed by the Carbon Trust and Fairtrade labels. The results obtained from the mixed logit models, which consider both the main effects and interaction terms between altruism score variables and sustainable labels, demonstrate that individuals’ altruistic orientation significantly influences their preferences and the associated price premiums for sustainable labels on coffee products. Notably, individuals who are selfless and willing to act for the well-being of others exhibit a stronger preference and valuation for fair trade and organic coffee.

These findings make valuable contributions to the existing literature on consumer choices regarding sustainable foods, particularly through the application of stated preference methods and a specific focus on both established and emerging environmentally friendly labels. Moreover, the study offers insights into how to enhance sustainable food consumption through targeting altruistic consumer subgroups and emphasizing the importance of fair trade and organic foods over newer green labels. However, it is worth noting that our survey was conducted during the pandemic period, which could potentially impact our empirical findings since consumer preferences for sustainable labels may also have been influenced.

## Figures and Tables

**Table 1:** Attributes and attribute levels.

Attributes	Levels
USDA organic label	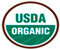
	No label
Fairtrade label	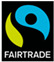
	No label
Carbon Trust label	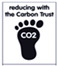
	No label
Price (USD per 12 oz)	4.95, 7.45, 9.95, 12.45

**Table 2:** Scale on altruism orientation.

Item	Description
1	I am willing to make sacrifices for the good of those around me
2	I enjoy contributing to charities and other non-profit organizations
3	Pay taxes is important because they fund programs such as schools and roads from which everyone benefits
4	I am comfortable receiving benefits even if I don’t contribute
5	My personal happiness is more important than the well-being of the average American

The order of the questions was randomized.

**Table 3:** Sample Description.

Variable	Definition	Mean
**Demographics**		
Female	1 = Yes, 0 = No	0.45 (0.50)
Age	Year	50.91 (16.91)
Household Income	1 =< USD 40K, 2 = USD 40K–USD 79K… to 5 => USD 160K	2.30 (1.14)
Education	0 =< College, 1 = College, 2 = Graduate	0.67 (0.74)
Household size	Person	3.47 (1.24)
Political view	1 = Liberal, 2 = Moderate, 3 = Conservative, 4 = Other	2.05 (0.77)
Religion	1 = Yes, 0 = No	0.77 (0.42)
White	1 = Yes, 0 = No	0.68 (0.47)
Employment	1 = Full time, 2 = Part time, 3 = Unemployed, 4 = Other	2.09 (1.04)
**Choice Frequency in the Choice Experiment**		
Opt-out option	0–8	1.128
		(0.105)
High-priced option	0–8	2.931
		(0.076)
Observations	Person	304

Note: numbers in parentheses are standard deviations.

**Table 4:** Preferences for sustainable coffee labels.

		(1)	(2)
**Mean**			
Price		−0.559 ***	−0.569 ***
		(0.024)	(0.024)
ASC_Opt-out_		−5.265 ***	−5.366 ***
		(0.219)	(0.224)
Organics		0.673 ***	0.582 ***
		(0.115)	(0.124)
Fairtrade		0.411 ***	0.372 ***
		(0.115)	(0.106)
Carbtrust		0.422 ***	0.367 ***
		(0.097)	(0.093)
Organics *Altruism			0.058 **
			(0.021)
Fairtrade * Altruism			0.061 ***
			(0.015)
Carbtrust * Altruism			0.029 *
			(0.019)
**Standard Deviation**			
Organics		1.603 ***	1.373 ***
		(0.122)	(0.193)
Fairtrade		1.428 ***	1.299 ***
		(0.119)	(0.142)
Carbtrust		1.135 ***	0.871 **
		(0.116)	(0.120)
**Model Statistics**			
Number of respondents		304	304
AIC		3449.764	3402.054
Loglikelihood		−1716.882	−1683.746

Note: numbers in parentheses are standard deviations. ***, **, and * indicate the 1%, 5%, and 10% significance levels, respectively.

**Table 5:** WTPs for sustainable coffee labels, USD/12 oz.

	Full Sample	Above Median Altruism Score	Below Median Altruism Score	*p*-Value
USDA Organics	1.204	1.482	1.053	<0.01
	(0.251)	(0.232)	(0.199)	
Fair trade	0.735	0.841	0.602	0.039
	(0.186)	(0.210)	(0.203)	
Carbon trust	0.715	0.820	0.633	0.084
	(0.145)	(0.161)	(0.175)	
Observations	304	148	156	

Note: numbers in parentheses are standard deviations. *p*-values are calculated using a *t*-test examining the equality of WTP between two samples.

## Data Availability

Available upon reasonable request.
